# Gastric Lanthanum Deposition in Advanced CKD: An Underrecognized Diagnostic Pitfall in Refractory Anemia and Mucosal Injury

**DOI:** 10.1155/ijne/2324899

**Published:** 2026-08-26

**Authors:** Yoshitaka Furuto, Daiki Yoshino, Iori Ejima, Sayako Ikeda, Akio Namikawa, Dai Sato, Yuko Shibuya

**Affiliations:** ^1^ Department of Hypertension and Nephrology, NTT Medical Center Tokyo, Shinagawa, Japan, ntt-east.co.jp

**Keywords:** chronic kidney disease, dialysis, gastric lanthanosis, lanthanum carbonate, refractory anemia

## Abstract

Lanthanum carbonate is a noncalcium phosphate binder used in advanced chronic kidney disease (CKD), especially in dialysis populations. Gastroduodenal lanthanum deposition is now a recognized histopathologic finding, but its clinical importance is heterogeneous. The central clinical question is not whether lanthanum can be found in the mucosa but when deposition should be interpreted as a possible contributor to mucosal injury and, otherwise, unexplained bleeding‐anemia phenotypes. Available evidence supports a spectrum from incidental histologic deposition to a smaller clinically meaningful subset associated with melena, occult or chronic upper gastrointestinal blood loss, iron deficiency, recurrent transfusion requirement, or erythropoiesis‐stimulating agent (ESA) hyporesponsiveness. Characteristic whitish lesions are useful endoscopic clues, but deposition can also occur in nonwhitish or minimally abnormal mucosa. Histopathology typically shows histiocyte‐rich accumulation of granular eosinophilic or light‐brown material, sometimes with a foreign‐body‐type reaction. A plausible subset‐specific pathway links mucosal deposition and macrophage activation to epithelial injury, chronic microbleeding, iron‐restricted erythropoiesis, and increased ESA requirements. Clinically meaningful disease is most plausible when exposure history, phenotype, mucosal context, biopsy findings, and—when observed—improvement after lanthanum withdrawal converge. Deposition alone does not establish causality, and the true prevalence of clinically meaningful disease remains unknown. Nevertheless, in selected patients with advanced CKD and refractory anemia, deliberate review of current and prior phosphate‐binder exposure, appropriate gastric biopsy even when endoscopy is nondiagnostic, and reassessment after discontinuation may reveal a potentially reversible diagnostic contributor.

**Summary**
• Gastric lanthanum deposition should be interpreted as a clinicopathologic spectrum. Many deposits are incidental or clinically silent, but in selected patients with advanced CKD—particularly those receiving dialysis—convergence of substantial lanthanum exposure, a compatible bleeding‐anemia phenotype, mucosal injury, and biopsy‐supported deposition may identify an underrecognized and potentially reversible contributor.

## 1. Introduction

Lanthanum carbonate is widely used as a noncalcium phosphate binder in patients with advanced chronic kidney disease (CKD) who require long‐term control of hyperphosphatemia. Since the first histopathologic reports in 2015, lanthanum deposition in the gastric and duodenal mucosa has become a recognizable clinicopathologic finding [1–7]. Early studies established that lanthanum can accumulate extensively within the lamina propria and produce distinctive histiocytic lesions [1, 2]. Surgical specimens have also demonstrated deposition in regional lymph nodes, confirming that the finding is neither a superficial artifact nor limited to endoscopic biopsy fragments [3].

The more important clinical question is how deposition should be interpreted. Clinicopathologic series have not established a consistent bleeding‐anemia phenotype; in one comparative study, measured blood‐test findings did not differ significantly between patients with and without gastric lanthanum deposition [4–6, 8, 9]. Conversely, case‐based reports describe upper gastrointestinal bleeding or iron deficiency anemia, with improvement after lanthanum withdrawal in several cases [10–13]. The available evidence, therefore, favors a spectrum rather than a binary disease model.

This distinction is especially relevant to nephrologists because anemia in advanced CKD is common and multifactorial. Iron deficiency, inflammation, inadequate erythropoiesis‐stimulating agent (ESA) response, nutritional deficiency, marrow disorders, dialysis‐related blood loss, and more familiar gastrointestinal sources are appropriately considered first. However, repeated escalation of iron or ESA therapy may delay recognition of an exposure‐associated mucosal lesion when the severity or persistence of anemia remains insufficiently explained.

This review reframes gastric lanthanum deposition as a contextual diagnostic problem. It compares incidental and clinically meaningful deposition, illustrates representative endoscopic and histopathologic appearances, proposes a biologically plausible bleeding‐anemia pathway, and provides a practical approach to suspicion, biopsy, withdrawal, and follow‐up. The strongest phenotype‐level evidence remains concentrated in dialysis populations and is derived largely from case reports and small clinicopathologic studies; conclusions should, therefore, remain cautious. Clinically informative reports are summarized in Supporting Table S1.

## 2. From Histologic Deposition to Clinical Relevance

Gastric lanthanum deposition is best interpreted along a clinicopathologic continuum (Table 1). At one end, lanthanum is detected microscopically in an asymptomatic patient without a demonstrated bleeding‐anemia phenotype or evidence that the lesion contributes to the clinical course. At the other, deposition occurs in a patient with substantial current or prior exposure; otherwise, unexplained melena or chronic blood loss, iron‐restricted erythropoiesis, recurrent transfusion requirement, or ESA hyporesponsiveness. Between these extremes are patients with visible whitish lesions or biopsy‐proven deposition but no demonstrable bleeding‐anemia phenotype [4–9].

**TABLE 1:** Practical comparison of incidental or clinically silent gastric lanthanum deposition and clinically meaningful deposition.

Domain	Incidental or clinically silent deposition	Clinically meaningful deposition
Symptoms	Deposition may be asymptomatic; in one seven‐patient series, five patients were asymptomatic and two had nonspecific gastrointestinal symptoms [6]. Other clinicopathologic studies did not establish a consistent bleeding‐anemia phenotype [4, 5, 8, 9].	Melena, suspected occult upper gastrointestinal blood loss, fatigue attributable to anemia, or recurrent bleeding episodes [10–13].
Exposure history	Current or prior exposure may be present; duration or cumulative dose alone does not establish clinical significance [4, 6, 8, 14].	Usually substantial current exposure in reported bleeding‐anemia cases [10–13]. Remote exposure may remain diagnostically relevant because deposition can persist for years after discontinuation, although persistence alone does not establish clinical causality [14].
Endoscopic findings	Classic whitish spots, annular or diffuse whitish mucosa may be present, or mucosa may appear nonwhitish; visible deposition can be clinically silent [5–8, 15–18].	Whitish lesions, erosion, reddish‐brown residue, subtle abnormalities, or apparently normal mucosa; concern increases when visible findings are insufficient to explain the clinical course [10–13].
Histopathology	Granular eosinophilic or light‐brown material within histiocytes, with variable or limited inflammatory response; deposition alone is not proof of injury [2–5, 8, 9, 19].	Biopsy‐proven deposition with prominent histiocytic or foreign‐body response and/or associated erosive, regenerative, or vulnerable mucosal background; clinical correlation remains essential [2, 10–12, 19].
Ancillary elemental confirmation	Usually unnecessary when morphology is typical and no compatible clinical phenotype is present; elemental analysis does not assign clinical significance in isolation [17, 20, 21].	SEM‐EDS may confirm lanthanum and phosphorus within the same deposit‐bearing area when morphology is atypical, competing inorganic deposits are considered, or diagnostic certainty is important; it supplements rather than replaces clinicopathologic correlation [17, 20, 21].
Clinical phenotype	A consistent bleeding‐anemia phenotype has not been established in available clinicopathologic series; one comparative study found no significant differences in measured blood‐test findings between deposition‐positive and deposition‐negative groups [4–6, 8, 9].	Iron restriction or iron deficiency, falling hemoglobin, recurrent transfusion requirement, increased ESA requirement, or, otherwise, unexplained refractory anemia [10–13].
Causality assessment	No convergent temporal or therapeutic‐response evidence links deposition to symptoms or anemia; morphology alone is insufficient [4–6, 8, 9].	Convergence of exposure, compatible phenotype, biopsy findings, exclusion of more likely causes, and—when present—improvement after withdrawal [10–12].
Biopsy strategy	Routine biopsy solely on the basis of exposure in an asymptomatic patient is not established; biopsy remains guided by clinical and endoscopic indications [7, 8].	Target whitish or erosive lesions and consider biopsy of nonclassic or normal‐appearing gastric mucosa when, otherwise, unexplained bleeding, iron deficiency or iron restriction, increasing ESA requirement, recurrent transfusion need, or refractory anemia sustains suspicion; consider duodenal sampling when gastric biopsies are negative or suspicion remains high [8, 12, 22].
Management	Deposition alone does not provide an evidence‐based indication for automatic discontinuation; management should be individualized in the absence of a compatible clinical phenotype [8, 23].	Consider withdrawal of lanthanum; maintain phosphate control with an alternative binder (e.g., sevelamer or, when appropriate, calcium carbonate); manage anemia or mucosal bleeding separately; and follow melena or other evidence of upper gastrointestinal bleeding, hemoglobin, iron status, transfusion need, and ESA dose. A clinically meaningful response is improvement in one or more of these outcomes. Scheduled endoscopic surveillance solely for lanthanum exposure is not supported; repeat endoscopy should be indication‐driven [10–12, 14].

*Note:* The categories represent ends of a continuum rather than validated diagnostic classes. Clinical relevance should be judged by convergence of exposure, phenotype, mucosal context, pathology, exclusion of competing causes, and response after withdrawal when available. Representative references are provided within each cell; the evidence base is primarily observational and case based.

No single feature establishes clinical relevance. Exposure duration and cumulative dose support plausibility but do not prove injury; whitish mucosa improves recognition but does not define pathogenicity; and histologic deposition alone may represent tissue retention without clinically important damage. A clinically meaningful interpretation becomes more persuasive when several elements converge: compatible exposure, a phenotype not adequately explained by routine evaluation, mucosal injury or a prominent histiocytic response, exclusion of more likely causes, and hematologic or bleeding improvement after withdrawal [10–12].

This framework avoids two opposing errors: overcalling every deposit as disease and dismissing all deposits as incidental. The first risks unnecessary discontinuation of an effective phosphate binder; the second may overlook a potentially reversible contributor to severe anemia or gastrointestinal bleeding.

## 3. Endoscopic and Histopathologic Clues

**FIGURE 1:**
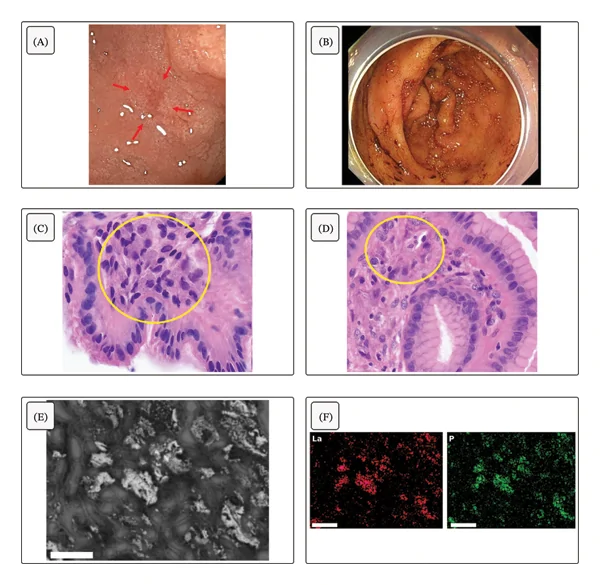
Representative endoscopic, histopathologic, and elemental findings in gastric lanthanum deposition. (A) Annular whitish gastric mucosa, a characteristic endoscopic pattern, adapted from Nishida et al. [8]. (B) Reddish‐brown residue and erosive change in a clinically meaningful bleeding‐anemia case, adapted from Furuto et al. [12]. (C, D) Gastric mucosa showing clusters of histiocytes containing light‐brown granular deposits, compatible with lanthanum deposition (hematoxylin and eosin staining; original magnification × 40), adapted from Furuto et al. [12]. (E) Backscattered scanning electron microscopy image showing bright electron‐dense deposits in gastric mucosa. (F) Corresponding SEM‐EDS elemental maps demonstrate lanthanum (La) and phosphorus (P) within the same deposit‐bearing area, providing direct elemental confirmation of lanthanum phosphate deposition. Panels E and F were adapted from Shinmura et al. [21] (original scale bar, 25 μm). All source articles are distributed under the Creative Commons Attribution 4.0 International License (https://creativecommons.org/licenses/by/4.0/). Source panels were cropped, resized proportionally, labeled, and arranged; no generative alteration, recoloring, or modification of the underlying scientific image content was performed.

Characteristic whitish gastric lesions remain an important clue. Reported patterns include discrete bright‐white spots, annular whitish mucosa, diffuse whitish mucosa, and white thickening along gastric folds [5–8, 15, 18]. In a clinicopathologic study, specific whitish features were associated with more extensive histiocytic infiltration although lanthanum was also detected in nonwhitish biopsy sites [8]. Representative appearances are shown in Figure 1.

Endoscopy is, therefore, informative but not definitive. Lanthanum deposition has been documented in nonwhitish biopsy sites and in patients without *Helicobacter pylori* infection [8, 16, 17]. In a bleeding‐anemia case, repeated examinations showed only erosion or no notable abnormality, yet random gastric biopsy demonstrated histiocytes containing granular deposits and the anemia resolved after lanthanum withdrawal [12]. A nondiagnostic examination should not terminate consideration when the exposure history and clinical phenotype remain strongly discordant with the visible findings.

Mucosal context modifies interpretation. Deposition is reported in atrophic, regenerative, metaplastic, and hyperplastic mucosa and may coexist with early gastric neoplasia [9, 24–27]. These backgrounds may increase susceptibility to deposition, complicate visual recognition, or provide competing explanations for mucosal injury. Duodenal involvement is also frequent and may be subtle, supporting duodenal biopsy when gastric findings are negative but suspicion remains high [22].

Histopathology is central to confirmation. The typical pattern is accumulation of fine granular eosinophilic or light‐brown material within macrophages or histiocytes in the lamina propria. More extensive lesions may show dense histiocytic infiltration, multinucleated giant cells, extracellular crystalline material, or a foreign‐body‐type reaction [2, 3, 10, 19]. Medication history is essential because the appearance can mimic other medication‐associated mucosal alterations or histiocytic disorders [19, 28]. When the identity of deposited material remains uncertain, scanning electron microscopy coupled with energy‐dispersive X‐ray spectroscopy (SEM‐EDS) can provide direct elemental confirmation by demonstrating lanthanum and phosphorus within the same deposit‐bearing area [17, 20, 21]. SEM‐EDS is particularly useful for atypical morphology or difficult differential diagnoses but should be regarded as a confirmatory method rather than a prerequisite for routine diagnosis [7, 21].

## 4. Proposed Mechanism of Clinically Meaningful Disease

**FIGURE 2:**
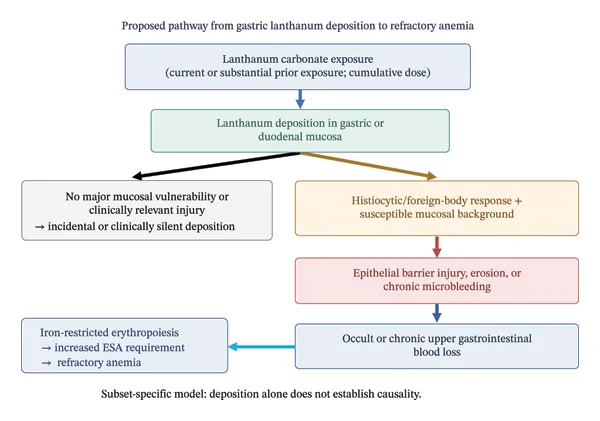
Proposed pathway from gastric lanthanum deposition to a bleeding‐anemia phenotype. Current or substantial prior lanthanum exposure can result in gastric or duodenal deposition. Many deposits remain clinically silent. In susceptible mucosa, histiocytic or foreign‐body response and epithelial barrier injury may promote erosion or chronic microbleeding, leading to occult upper gastrointestinal blood loss, iron‐restricted erythropoiesis, increased erythropoiesis‐stimulating agent requirements, and refractory anemia. This is a biologically plausible, subset‐specific model rather than proof of universal causality [2, 9–12, 29, 30].

The mechanistic link between deposition and refractory anemia should be regarded as a biologically plausible, subset‐specific model rather than a universally established causal pathway (Figure 2). Lanthanum exposure permits tissue deposition; whether that deposition remains clinically silent or becomes injurious likely depends on cumulative exposure, mucosal susceptibility, tissue handling, and the intensity of the local host response.

Histologic reports consistently describe macrophage or histiocyte accumulation and foreign‐body‐type reaction [2, 10, 19]. Experimental work using a Caco‐2/THP‐1 coculture model suggests that lanthanum deposition can activate macrophages and indirectly impair epithelial barrier integrity [29]. Regenerative change, intestinal metaplasia, and foveolar hyperplasia have been associated with greater deposition, supporting the possibility that pre‐existing mucosal vulnerability modifies the response [9]. Observations concerning macrophage polarization and clearance further suggest that local tissue handling may influence persistence and severity [30].

In a susceptible patient, epithelial barrier injury, erosion, or low‐grade mucosal bleeding could produce chronic upper gastrointestinal blood loss. Because fecal immunochemical testing may have reduced sensitivity for upper gastrointestinal bleeding after degradation of globin during intestinal transit, chronic microbleeding may remain clinically occult [31]. Progressive iron restriction may then increase ESA requirements and contribute to transfusion dependence or refractory anemia.

This pathway is supported by convergence rather than by definitive comparative trials. Many deposits are asymptomatic, and substantial deposition can persist long after discontinuation [14]. Accordingly, neither the presence of histiocytes nor a high cumulative dose should be equated automatically with disease. Improvement after withdrawal strengthens causal inference but does not eliminate the need to reassess competing causes.

## 5. Diagnostic Pitfalls in Advanced CKD With Refractory Anemia

The first pitfall is failure to revisit the diagnosis when anemia treatment is intensified repeatedly. Persistent anemia despite adequate iron and ESA therapy should prompt renewed assessment of inflammation, nutrition, hemolysis, marrow disease, dialysis‐related losses, and gastrointestinal bleeding rather than automatic dose escalation alone.

The second pitfall is false reassurance from endoscopic appearance. Classic white lesions increase recognition, but clinically relevant deposition may occur with erosion, subtle mucosal change, or apparently normal mucosa [8, 12, 16, 17]. When the clinical course is disproportionate to the visible findings, targeted biopsy of abnormal sites and additional biopsy of nonclassic or normal‐appearing mucosa may be justified.

The third pitfall is incomplete medication history. Both current and substantial prior lanthanum exposure matter. Dose and cumulative intake are associated with deposition [4, 8], and gastric lanthanosis has remained detectable years after the drug was stopped [14]. Phosphate‐binder history should, therefore, be reviewed with the same rigor applied to antiplatelet agents, anticoagulants, nonsteroidal anti‐inflammatory drugs, and other medications associated with gastrointestinal injury.

The fourth pitfall is overreliance on negative stool testing. A negative fecal immunochemical test should not provide false reassurance or preclude further evaluation when an upper gastrointestinal bleeding phenotype remains clinically plausible [31].

## 6. When to Suspect and How to Confirm

Suspicion should be highest when three domains converge: (1) advanced CKD with current or substantial prior lanthanum exposure; (2) a refractory or otherwise insufficiently explained bleeding‐anemia phenotype, including melena, iron restriction, recurrent transfusion requirement, or ESA hyporesponsiveness; and (3) gastrointestinal findings that are absent, subtle, or inadequate to explain the severity of the clinical course.

Routine anemia evaluation remains essential. Iron indices, inflammatory markers, nutritional status, hemolysis, marrow‐related disorders when indicated, dialysis adequacy, access‐related blood loss, and common gastrointestinal causes should be assessed first. Gastric lanthanum deposition should be considered an additional diagnosis, not a substitute for this workup.

When suspicion remains sufficient, biopsy strategy should not be restricted to classic white lesions. Targeted biopsy of whitish or erosive mucosa is appropriate, but biopsy of nonwhitish or normal‐appearing gastric mucosa may be diagnostic when the phenotype is compelling [8, 12]. Because duodenal deposition is common, duodenal sampling should be considered if gastric biopsies are negative or if duodenal abnormalities are present [22]. Pathologists should be informed of lanthanum exposure so that granular histiocytic deposits are interpreted in the correct clinical context.

A diagnosis of clinically meaningful deposition should be framed probabilistically. The most persuasive combination is biopsy‐proven deposition plus a compatible phenotype, no more plausible alternative explanation, and improvement after withdrawal. The absence of improvement should prompt renewed evaluation rather than an assumption that persistent deposits continue to be the dominant cause.

### 6.1. Practical Phenotype‐Driven Summary

Upper endoscopy with biopsy should be considered in patients with advanced CKD and current or substantial prior lanthanum exposure when, otherwise, unexplained melena, iron deficiency or iron restriction, increasing ESA requirement, recurrent transfusion need, or refractory anemia persists after standard evaluation. Biopsy should target whitish or erosive lesions, but additional sampling of nonwhitish or normal‐appearing gastric mucosa is reasonable when the phenotype remains compelling. Duodenal sampling may be added when gastric biopsies are negative, duodenal abnormalities are present, or clinical suspicion remains high. Discontinuation of lanthanum is most strongly supported when biopsy‐proven deposition coexists with a compatible bleeding‐anemia phenotype and no more plausible cause, particularly when mucosal injury is present or the phenotype is severe or worsening despite appropriate supportive care.

## 7. Management and Follow‐Up

When gastric lanthanum deposition is considered clinically meaningful, withdrawal of lanthanum carbonate is the principal exposure‐directed intervention. Withdrawal functions as both treatment and a diagnostic test. A clinically meaningful response may include resolution of melena or other evidence of upper gastrointestinal bleeding, recovery or stabilization of hemoglobin, reduced transfusion requirement, improvement in iron status, or a lower ESA requirement [10–12]. No fixed follow‐up interval can be recommended from the current evidence; response should be judged from the trajectory of these outcomes relative to the pretreatment course and concurrent interventions.

Phosphate control should be maintained with an alternative binder or strategy selected according to calcium balance, pill burden, gastrointestinal tolerance, vascular calcification risk, and the patient’s broader CKD‐mineral and bone disorder profile. Common alternatives include sevelamer or, when appropriate, a calcium‐based binder such as calcium carbonate, depending on local availability and patient factors. Replacement of the phosphate binder should be distinguished from supportive treatment of associated anemia or mucosal bleeding, which may include iron replacement, ESA adjustment, transfusion when clinically required, and treatment of coexisting mucosal lesions. The presence of lanthanum deposition does not imply that all exposed patients require discontinuation; asymptomatic deposition without a compatible phenotype should be managed individually rather than automatically labeled as adverse disease. This localized, phenotype‐driven approach should be distinguished from the generally favorable systemic safety and tolerability profile of lanthanum carbonate [23].

Follow‐up should document hemoglobin trajectory, transfusion exposure, iron indices, ESA dose, melena or other bleeding symptoms, and the need for further gastrointestinal evaluation. Histologic deposits may persist after withdrawal [14], so clinical improvement is more informative than immediate morphologic disappearance. Current evidence is insufficient to recommend scheduled endoscopic surveillance solely because a patient is receiving or has previously received lanthanum carbonate. Repeat endoscopy and biopsy should remain indication‐driven, for example, when bleeding persists, the diagnosis remains uncertain, or another mucosal disorder requires surveillance; routine serial biopsy solely to prove clearance is not supported by current evidence.

Failure to improve after withdrawal should trigger reconsideration of competing or coexisting causes. Advanced CKD anemia is often multifactorial, and lanthanum deposition may be contributory without being sufficient to explain the entire phenotype.

### 7.1. Practical Management Summary

In a patient with a compatible phenotype and biopsy‐proven deposition, discontinue lanthanum, maintain phosphate control with an alternative binder, treat anemia or mucosal bleeding separately, and follow clinical and hematologic response rather than histologic clearance. Lack of improvement should prompt reassessment of competing causes rather than scheduled surveillance for lanthanum deposition alone.

## 8. Evidence Limitations and Research Priorities

The evidence base is limited by small samples, retrospective designs, selection of patients who underwent endoscopy and biopsy, heterogeneity in pathology definitions, and publication bias toward unusual or severe cases. No validated diagnostic criteria distinguish incidental from clinically meaningful deposition, and the prevalence of bleeding‐anemia phenotypes among all exposed patients is unknown.

Future studies should prospectively characterize cumulative exposure, endoscopic phenotype, standardized histologic burden, mucosal inflammatory response, iron indices, ESA requirements, and outcomes after withdrawal. A clinically useful case definition will probably require integration of exposure, phenotype, pathology, and response rather than reliance on any single morphologic feature.

## 9. Conclusion

Gastric lanthanum deposition is a recognizable clinicopathologic lesion with variable clinical relevance. Many deposits are incidental or clinically silent, but a clinically meaningful subset may contribute to mucosal injury, occult or overt upper gastrointestinal bleeding, iron restriction, and ESA‐refractory anemia. Clinical relevance is most plausible when substantial exposure, a compatible phenotype, mucosal context, biopsy‐supported deposition, and improvement after withdrawal converge.

For nephrologists, the practical lesson is to maintain diagnostic awareness without overcalling causality. In selected patients with advanced CKD and, otherwise, unexplained refractory anemia, the review of current and prior phosphate‐binder exposure, biopsy even when endoscopy is nondiagnostic, lanthanum withdrawal, and structured hematologic follow‐up may identify a potentially reversible contributor.

## Funding

No funding was received for this work.

## Ethics Statement

The authors have nothing to report.

## Consent

The authors have nothing to report.

## Conflicts of Interest

The authors declare no conflicts of interest.

## Supporting Information

Additional supporting information can be found online in the Supporting Information section.

## Supporting information


**Supporting Information** Supporting Table S1. Clinically informative reports of gastric lanthanum deposition in CKD, with emphasis on bleeding‐anemia phenotypes and diagnostic implications.

## Data Availability

Data sharing is not applicable because no datasets were generated or analyzed in this review.
