# Supplementary material for: Gastric Lanthanum Deposition in Advanced CKD: An Underrecognized Diagnostic Pitfall in Refractory Anemia and Mucosal Injury
Source: Int J Nephrol. 2026 Aug 26;2026:2324899. doi: 10.1155/ijne/2324899 (PMC13509423; doi:10.1155/ijne/2324899)
Supplement: Supplementary file 1 [file IJNE-2026-2324899-s001.docx]

**Supplementary Table S1. Clinically informative reports of gastric lanthanum deposition in CKD, with emphasis on bleeding-anemia phenotypes and diagnostic implications.**

| **Study** | **Design / population** | **Exposure / setting** | **Endoscopic findings** | **Histopathology** | **Bleeding / anemia relevance** | **Practical implication** |
| --- | --- | --- | --- | --- | --- | --- |
| Makino et al., 2015 [1] | Histopathologic case report; peritoneal dialysis | Lanthanum-treated advanced CKD | Gastric lesion recognized endoscopically | Extensive gastric mucosal deposition | Not phenotype-defining | Foundational proof of a true clinicopathologic lesion. |
| Haratake et al., 2015 [2] | Pathology report; dialysis patients | Chronic lanthanum exposure | Not the principal emphasis | Massive deposition with peculiar histiocytic lesions | Indirect support | Established foreign-body-type mucosal reaction. |
| Yabuki et al., 2016 [3] | Surgical pathology series; 3 dialysis patients with gastric cancer | Lanthanum-treated dialysis | Complex neoplastic mucosal background | Gastroduodenal and regional lymph-node deposition | Indirect support | Shows deeper distribution and coexistence with major background pathology. |
| Goto and Ogawa, 2016 [4] | Clinicopathologic study; 13 gastric cases | Dialysis patients receiving lanthanum | Deposition recognized in exposed patients | Frequent histologic deposition | No uniform bleeding phenotype | Supports routine diagnostic awareness but not universal pathogenicity. |
| Shitomi et al., 2017 [5] | Clinicopathologic series; 23 dialysis patients | Lanthanum-treated dialysis | Reflective bright-white lesions | Eosinophilic histiocytes | Bleeding/anemia not the main focus | Defines a recognizable clinicopathologic pattern. |
| Murakami et al., 2017 [6] | Retrospective endoscopic series; 7 patients | Ongoing exposure for 5–45 months (median, 27 months) | All 7 had whitish lesions; lesions progressed in 3 of 5 patients with serial endoscopy | Pathology-confirmed deposition | Five patients were asymptomatic; pathogenicity remained uncertain | Defines the endoscopic spectrum and documents progression during continued exposure without establishing universal clinical injury. |
| Valika et al., 2016 [8] | Case report; dialysis patient with anemia and upper GI bleeding | Long-term lanthanum exposure (4 years) | Nodular upper gastrointestinal lesion | Foreign-body reaction with lanthanum-compatible deposits | Direct bleeding phenotype; bleeding ceased after withdrawal | Supports a clinically significant bleeding subset and therapeutic response after withdrawal. |
| Awad et al., 2019 [9] | Case report; ESRD with biopsy-proven upper GI deposition | Lanthanum exposure | No overt alternative bleeding source | Biopsy-proven deposition | Iron deficiency anemia; improved after withdrawal | Supports occult or underrecognized blood loss in selected patients. |
| Nishida et al., 2020 [10] | Two-part retrospective clinicopathologic study; 42 hemodialysis patients and 51 biopsy specimens from 23 deposition-positive patients | Cumulative exposure assessed | 27 whitish and 24 non-whitish biopsy sites contained deposition | Whitish features were associated with greater histiocytic infiltration; total dose was higher in deposition-positive patients | No significant difference in measured blood-test findings between deposition-positive and deposition-negative groups | Demonstrates an exposure association, frequent non-whitish deposition, and the absence of a uniform hematologic consequence. |
| Ban et al., 2017 [11] | Histopathologic susceptibility study | Exposed dialysis patients | Mucosal background emphasized | Deposition associated with regenerative change, intestinal metaplasia, and foveolar hyperplasia | Indirect support | Suggests mucosal vulnerability modifies deposition and possibly clinical expression. |
| Furuto et al., 2025 [12] | Case report; hemodialysis with recurrent severe anemia and melena | 1500 mg/day for approximately 3 years | Repeatedly nondiagnostic; no classic white lesions at fourth gastroscopy | Random biopsy showed histiocyte clusters with light-brown granular deposits | Direct bleeding-anemia phenotype; anemia and melena resolved after withdrawal | Shows that clinically meaningful disease can occur despite nonspecific endoscopy and negative stool testing. |
| Martínez-Acitores de la Mata et al., 2021 [28] | Case report | Lanthanum exposure in advanced kidney failure | Massive upper gastrointestinal bleeding | Gastric lanthanosis documented | Direct severe bleeding phenotype | Supports the possibility of acute, clinically consequential hemorrhage; outcome data are not used to infer a general treatment effect. |
| Ullmann and Erbersdobler, 2021 [27] | Case report; prior exposure | Lanthanum stopped 7 years earlier | Persistent lesion on follow-up | Persistent gastric lanthanosis | No bleeding-anemia causality was established; symptoms were attributed to another condition in the source report. | Shows persistence 7 years after discontinuation; remote exposure can remain diagnostically relevant, but persistence alone does not establish clinical significance. |
| Iwamuro et al., 2019 [29] | Retrospective observational study; pathologically proven gastrointestinal deposition | Lanthanum-treated patients undergoing upper gastrointestinal biopsy | White villi were present in 15 of 17 deposition-positive patients (88.2%); 2 of 17 had no white villi | Duodenal deposition was detected in 17 of 19 patients (89.5%) who underwent duodenal biopsy | Not a bleeding-anemia outcome study | Supports duodenal biopsy even when white villi are absent. |
| Shinmura et al., 2020 [31] | Diagnostic method study; 9 H&E-stained upper gastrointestinal slides from 6 dialysis patients | All patients had dialysis and lanthanum exposure | Endoscopic phenotype was not the primary study endpoint | All 9 slides showed La and P accumulation in the brown-pigment area by NanoSuit-CLEM SEM-EDS; bright-area La and P were significantly higher than background (both p<0.001) | Not a bleeding-anemia outcome study | Provides direct elemental confirmation and supports SEM-EDS as an ancillary method when morphology is atypical or uncertain. |

Footnote: Case reports provide the most direct evidence for bleeding-anemia phenotypes and response after withdrawal, whereas clinicopathologic studies define lesion recognition, morphologic range, exposure associations, persistence, and mucosal context. Diagnostic-method studies establish the role of SEM-EDS for elemental confirmation. Evidence levels are not equivalent across study designs, and absence of a reported outcome should not be interpreted as evidence of no effect.
